# Basic Blue Dye Adsorption from Water Using Polyaniline/Magnetite (Fe_3_O_4_) Composites: Kinetic and Thermodynamic Aspects

**DOI:** 10.3390/ma12111764

**Published:** 2019-05-30

**Authors:** Amir Muhammad, Anwar-ul-Haq Ali Shah, Salma Bilal, Gul Rahman

**Affiliations:** 1 Institute of Chemical Sciences, University of Peshawar, Peshawar 25120, Pakistan; amirics2015@gmail.com (A.M.); gul_rahman47@uop.edu.pk (G.R.); 2 National Centre of Excellence in Physical Chemistry, University of Peshawar, Peshawar 25120, Pakistan; 3 TU Braunschweig Institute of Energy and Process Systems Engineering, Franz-Liszt-Straße 35, 38106 Braunschweig, Germany

**Keywords:** Basic Blue 3 dye (BB3), polyaniline/Fe_3_O_4_ composite, Freundlich, Langmuir, Tempkin and Dubbanin-Radushkavitch adsorption isotherm

## Abstract

Owing to its exciting physicochemical properties and doping–dedoping chemistry, polyaniline (PANI) has emerged as a potential adsorbent for removal of dyes and heavy metals from aqueous solution. Herein, we report on the synthesis of PANI composites with magnetic oxide (Fe_3_O_4_) for efficient removal of Basic Blue 3 (BB3) dye from aqueous solution. PANI, Fe_3_O_4_, and their composites were characterized with several techniques and subsequently applied for adsorption of BB3. Effect of contact time, initial concentration of dye, pH, and ionic strength on adsorption behavior were systematically investigated. The data obtained were fitted into Langmuir, Frundlich, Dubbanin-Rudiskavich (D-R), and Tempkin adsorption isotherm models for evaluation of adsorption parameters. Langmuir isotherm fits closely to the adsorption data with R^2^ values of 0.9788, 0.9849, and 0.9985 for Fe_3_O_4_, PANI, and PANI/Fe_3_O_4_ composites, respectively. The maximum amount of dye adsorbed was 7.474, 47.977, and 78.13 mg/g for Fe_3_O_4_, PANI, and PANI/Fe_3_O_4_ composites, respectively. The enhanced adsorption capability of the composites is attributed to increase in surface area and pore volume of the hybrid materials. The adsorption followed pseudo second order kinetics with R^2^ values of 0.873, 0.979, and 0.999 for Fe_3_O_4_, PANI, and PANI/Fe_3_O_4_ composites, respectively. The activation energy, enthalpy, Gibbs free energy changes, and entropy changes were found to be 11.14, −32.84, −04.05, and −0.095 kJ/mol for Fe_3_O_4_, 11.97, −62.93, −07.78, and −0.18 kJ/mol for PANI and 09.94, −74.26, −10.63, and −0.210 kJ/mol for PANI/Fe_3_O_4_ respectively, which indicate the spontaneous and exothermic nature of the adsorption process.

## 1. Introduction

The use of organic synthetic dyes has increased dramatically and uncontrollably in last few decades. Different types of dyes are frequently employed in plastics, paper, cosmetics, leather, and textile industries for coloring purposes [1,2,3]. These dyes are released in water as effluents, which are of low biological oxygen demand (BOD) and high chemical oxygen demand (COD) [4]. Some of these dyes, such as azo-dyes, are toxic and carcinogenic in nature. Their addition into nearby streams and rivers contaminates water and greatly upsets the biological activities of aquatic life [5,6]. It is highly desirable to explore efficient technologies for remediation and separation of these potential pollutants from effluents.

Various protocols and techniques, such as reverse osmosis, precipitation, coagulation, membrane filtration, chemical oxidation, electrochemical methods, ion exchange, and adsorption are used to remove these dyes and other hazardous materials from polluted water [7,8]. However, adsorption is the most frequently used technique to remove dyes from water, because this technique, in addition to easiness and low cost, causes low generation of residues and the adsorbent used may be regenerated and reused [9,10,11]. Several adsorbents, such as rice husk, sawdust, activated carbon, orange peel, and chitosan, have been used to remove dyes from aqueous environment [12,13,14,15]. However, the major drawback of the use of these materials is that they must be activated either physically or chemically before use. Physically these adsorbents are usually activated at very high temperature, which needs high energy. After removal of dyes, desorption must be carried out to regenerate the adsorbent, which is sometimes complicated and mostly generates secondary pollutants [16], while if thrown without treatment, they will cause water pollution. These complications make the use of these materials very expensive and time consuming, and threatening to the environment. Although activated carbon has been known as the most efficient adsorbent owing to its high specific surface area, its use is also restricted due to the non-selectivity and regeneration issues. Therefore, there is a need for the development of an environment-friendly material that is easy to regenerate [17]. 

In recent years, some conducting polymers, such as polyaniline, polythiophene, polypyrrole, and their composites with other materials have attracted much interest because of their conducting behavior and fascinating physicochemical properties. Such materials have been successfully applied in solar cells, fuel cells, sensors, super-capacitors, and for corrosion protection in organic coating [18,19,20]. Polypyrrole/TiO_2_, polypyrrole/graphene oxide/Fe_3_O_4_, and polyaniline/magnetite have also been applied as adsorbents to remove dyes and heavy metals from aqueous environments [21,22,23]. Polyaniline, which exists in various oxidation states, is environmentally stable and a good conducting material with excellent electrochemical properties and can be easily prepared with less cost [24,25,26]. PANI and its composites with other materials, such as TiO_2_, MnO_2_, Fe_2_O_3,_ SeO_2_, SiO_2_, Ag, Cd, and Zn, have been applied in sensors, biosensors, rechargeable batteries, fuel cells, and solar cells [27,28,29,30,31]. Some of these composites have also been used as adsorbents to remove heavy metals and dyes from aqueous environments [32,33]. Janaki et al. [34] removed Coomassie brilliant blue, congo red, and methylene blue from aqueous solution using polyanilline/chitosan composites. Sultana et al. [35] synthesized copper ferrite nanoparticles doped polyanilline for removal of direct yellow-27 from aqueous solution. Ayad and Al-Naser [1] applied polyanilline nanotube base as an adsorbent to remove methylene blue from an aqueous environment. 

Magnetic materials such as Fe_3_O_4_ have attracted special attraction from scientists because of their numerous applications, such as in drug delivery systems [36], magnetic resonance imaging (MRI) [37,38], efficient hyperthermia for removal of cancer [39], clinical diagnosis [40], and removal of heavy metals form aqueous solution [41,42]. Fe_3_O_4_ can be prepared by a number of methods, including hydrothermal method [43], chemical co-precipitation method [44], sol-gel [45], gas phase [46], liquid phase [47], and micro emulsion methods [48]. Polyaniline/magnetite(Fe_3_O_4_) composites have the advantage of being stable at high temperatures and can be synthesized easily from low cost materials, which make them superior over the other existing natural/synthetic and biodegradable polymers for the adsorption of dyes. They can be regenerated easily after adsorption and due to their conductive nature, electrochemical study of these materials after adsorption can be carried out. Several reports are available on the use of PANI/iron-oxide-based materials as adsorbents for dyes; a comparison of adsorption properties of these materials with the present work is made in Table S1 of Supplementary Information.

The present study is aimed at investigating the adsorption capacity of Fe_3_O_4_ and PANI by synthesizing PANI/Fe_3_O_4_ composites for the removal of Basic blue 3 dye from aqueous solution. For comparison, PANI and Fe_3_O_4_ were also synthesized and tested for dye removal efficiency. Chemical co-precipitation protocol was adopted for the preparation of Fe_3_O_4_ in basic medium in the temperature range of 85–90 °C. PANI and PANI/Fe_3_O_4_ composites were synthesized by chemical oxidation method using FeCl_3_ as an oxidant. The synthesized Fe_3_O_4,_ PANI and composites were characterized with Fourier transforms infrared spectroscopy (FTIR), scanning electron microscopy (SEM), X-ray diffraction (XRD), energy Dispersive X-Ray spectroscopy (EDX), and surface area measurements. Batch adsorption experiments were carried out to study the effect of pH, initial concentration of dye, contact time, and temperature on the adsorption phenomenon by using UV-Visible spectrophotometer. The resulted data were fitted into Friundlich, Langmuir, Tempkin, and The Dubinin-Radushkevitch (D-R) adsorption models. Kinetics and thermodynamic aspects of the adsorption of Basic blue 3 dye on these materials were also investigated. 

## 2. Experimental

### 2.1. Materials 

Aniline (Across) was distilled before use under vacuum. Basic blue 3 dye, FeCl_3_·6H_2_O (Sigma-Aldrich, St. Louis, MO, USA), FeSO_4_·7H_2_O (Merck, Kenilworth, NJ, USA), Na_2_SO_4_ (Panreac Quimica SA, Barcelona, Spain), and Dodecyl benzene sulphonic acid, DBSA, (Across) were used as received. All chemicals used were of analytical grade.

### 2.2. Synthesis of PANI

PANI was synthesized via chemical oxidation method by adding 0.3 mol (0.82 mL) aniline in 30 mL double distilled water. Then, 0.02 mol (0.25 mL) Do-decylbenzene sulphonic acid (DBSA) prepared in 40 mL double distilled water was added as an emulsifying agent as well as a dopant. Afterwards, 0.01 M FeCl_3_. 6H_2_O solution (30 mL) was added dropwise to this mixture as an oxidant. The solution was stirred on a magnetic stirrer for about 12 h. Initially, the solution was a milky white color, but after an hour the solution turned light green and then dark green after 3 hours. Finally, the product was extensively washed with acetone and double distilled water till the filtrate became clear and dried in an oven at 60 °C for 24 h.

### 2.3. Synthesis of Fe_3_O_4_

**Scheme 1:**
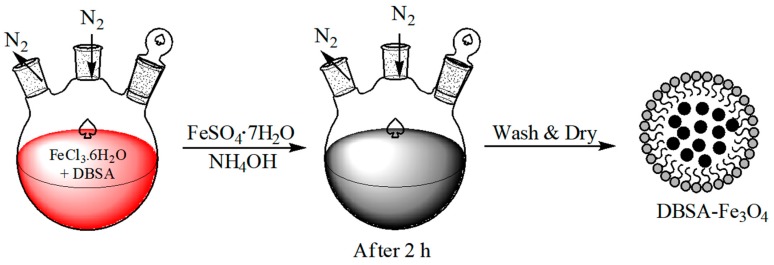
Synthesis of Fe_3_O_4_.

Chemical co-precipitation method was used to synthesize Fe_3_O_4_ by mixing FeCl_3_·6H_2_O and FeSO_4_·7H_2_O in a molar ratio of 2:0.5. DBSA was used as the emulsifying agent. The reaction was performed in basic medium (pH 10) in the temperature range of 85–90 °C. Then, 5 M ammonia solution (60 mL) was added as precipitating agent, which turned the reaction mixture black. The mixture was continuously stirred for about 2 h. Then, it was washed with plenty of distilled water and ethanol until the filtrate became clear. The resulting black precipitate was dried in an oven at 80 °C for 10 h and finally annealed in a furnace at 600 °C for 5 h [49]. The schematic representation of the process is presented in Scheme 1.

### 2.4. Synthesis of PANI/Fe_3_O_4_ Composites

**Scheme 2:**
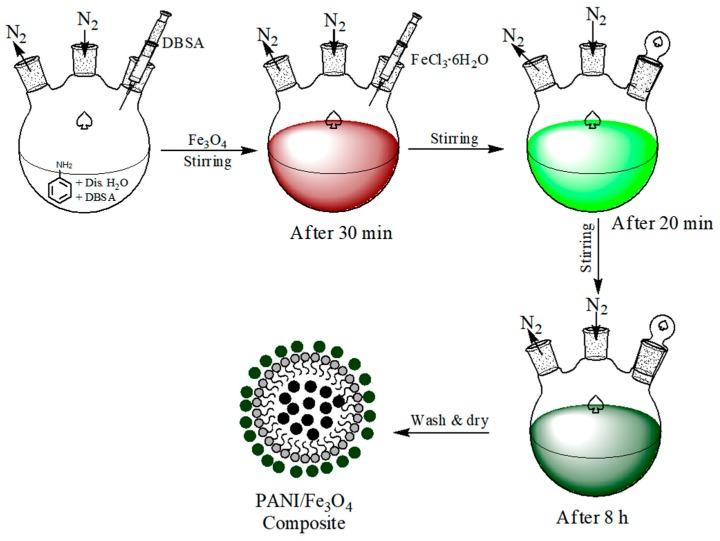
Synthesis of PANI/Fe_3_O_4_ Composite.

Chemical oxidation method was used to synthesize PANI/Fe_3_O_4_ composites. First, 0.2 g Fe_3_O_4_ was mixed with 1.818 mL of aniline suspended in double distilled water (50 mL) and DBSA (0.5 mL). The mixture was stirred for about 30 min and followed by addition of 0.15 M FeCl_3_·6H_2_O prepared in 40 mL double distilled water as oxidizing agent. Initially the reaction mixture was milky white due to DBSA but turned reddish brown after addition of Fe_3_O_4_ particles. When oxidant was added a light green color appeared within 20 min, which changed into dark black after about 2 h. After 8 h continuous stirring, the synthesized product was washed with acetone and plenty of double distilled water. Finally, the clean precipitate was dried in an oven at 60 °C for 24 h. The schematic representation of the process in provided in Scheme 2.

#### 2.4.1. Batch Adsorption Study for Removal of BB3 Dye

Basic blue 3 dye solution of desired concentrations ranging 0.01–110 (mg/L) were prepared in 20 mL volume by dilution method from the respective stock solution. To these solutions, Fe_3_O_4_ was added and shacked in a shaker at a speed of 150 rpm for 90 min. These solutions were then filtered and the concentration of dye was determined using a carry-50 UV-Visible spectrophotometer. The amount of dye adsorbed was determined by using the following equation [50].

(1) $$q_{e}=\frac{{(C}_{i}{-C}_{e})}{m}\times V$$

where q_e_ (mg/g) is the amount of dye adsorbed at equilibrium, Ci and Ce are the initial concentration and the concentration of dye present at equilibrium, respectively, m (g) is the amount of adsorbent added, and V (L) is the volume of solution. The effects of contact time, pH, initial concentration of dye, temperature, and ionic strength on the adsorption process were studied. The data obtained were used to calculate the kinetics and thermodynamic parameters. The same procedure was adopted for studying adsorption of Basic blue 3 dye on PANI and PANI/Fe_3_O_4_ composites_._

After adsorption of BB3 dye on PANI/Fe_3_O_4_ composite, it was collected in filter paper with plenty of double distilled water to run out the adsorbed dye. After removal of BB3, the PANI/Fe_3_O_4_ composite was washed with 0.1 M HCl, to remove the remaining dye from the surface. In this way composites were regenerated and could be reused

#### 2.4.2. Characterization

The surface morphologies of Fe_3_O_4_, PANI, and PANI/Fe_3_O_4_ composites were studied with scanning electron microscopy (SEM) using a JSM-6490 (JEOL, Tokyo, Japan) electron microscope. FTIR spectra of the Fe_3_O_4_, PANI, and Fe_3_O_4_/PANI composites were recorded with IRAffinity-1S Shimadzu Fourier Transform Infrared Spectrophotometer (Shimadzu, Tokyo, Japan) in the spectral range of 400 to 4000 cm^−1^. X-ray diffraction (XRD) were recorded with by using Cu Kα radiations (λ = 1.5405 Å) through JEOL JDX-3532 (JEOL, Tokyo, Japan). UV-Visible spectrophotometer (Perkin Elmer, Buckinghamshire, UK) was used to find out the concentration of dye in the solution and to check the amount of dye adsorbed on the composite. Energy-dispersive X-ray (EDX) spectrophotometer model (Oxford, UK) Inca 200 was used for determination of elemental composition. BET surface areas of PANI, Fe_3_O_4_, and composite before and after adsorption were determined in N_2_ atmosphere by adsorption–desorption method with surface area analyzer model 2200 e Quanta Chrome (Boynton Beach, FL, USA).

## 3. Results

### 3.1. Characterization

After synthesis, different techniques were used in order to know about the structural and morphological features and to get insights into the formation of composites and their adsorption properties. For comparison purposes, the same studies were carried out in parallel for Fe_3_O_4_ and PANI alone.

#### 3.1.1. SEM Study

**Figure 1:**
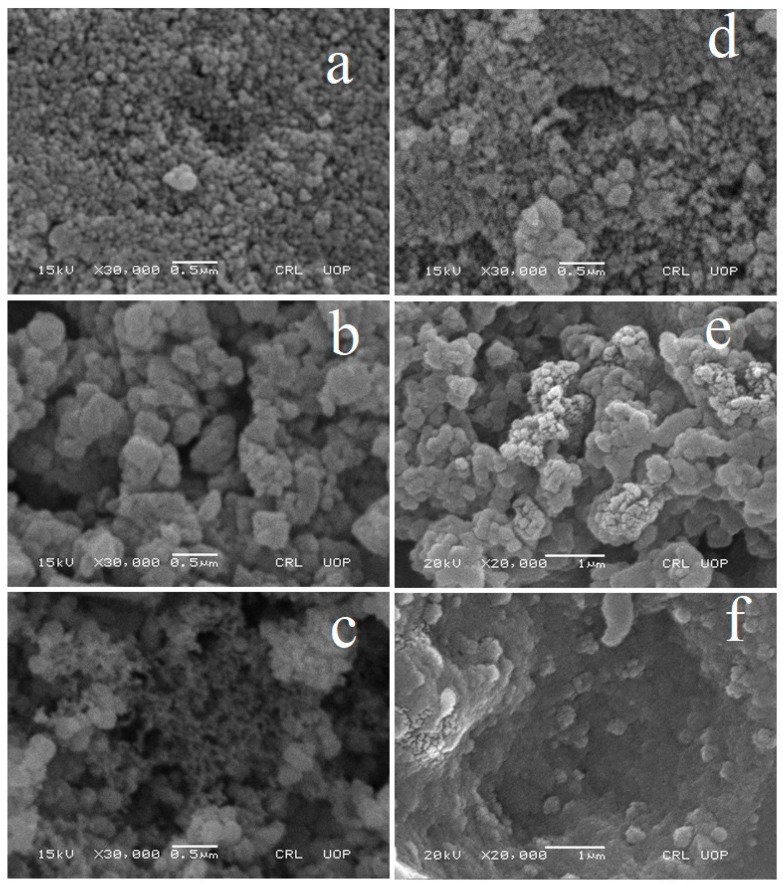
SEM images of Fe_2_O_3_, PANI, and PANI/Fe_2_O_3_ composites before (**a**,**c**,**e**) and after (**b**,**d**,**f**) adsorption of BB3.

The surface morphology of Fe_3_O_4_, PANI, and PANI/Fe_3_O_4_ composites were studied with scanning electron microscopy. The SEM image (Figure 1a) shows that Fe_3_O_4_ consists of finite spherical shape with average particle size of 0.25 µm, which tends to form aggregates. It is somewhat porous in texture and becomes rough after adsorption of BB3 (Figure 1b). The adsorption of dye on the surface of Fe_3_O_4_ decreases its porosity, as reported elsewhere [51]. Shreepathi and Holze reported fibrous morphology of PANI prepared in different concentrations of DBSA [52]. The SEM image of PANI synthesized in this work shows cauliflower-like surface morphology, which after adsorption of dye changes into clusters of small ball-like structures, shown in Figure 1c,d. The SEM image of PANI/Fe_3_O_4_ depicts surface characteristics of both PANI and Fe_3_O_4_. Close observation of the composite morphology indicates adherence of Fe_3_O_4_ particles on the surface of PANI. The average size of composite particles was 0.28 µm. The development of magnetic micro and nanoparticle composites with PANI has been reported to greatly enhance adsorption characteristics of the hybrid materials [53,54,55,56].

#### 3.1.2. UV-Vis Spectroscopic Study

**Figure 2:**
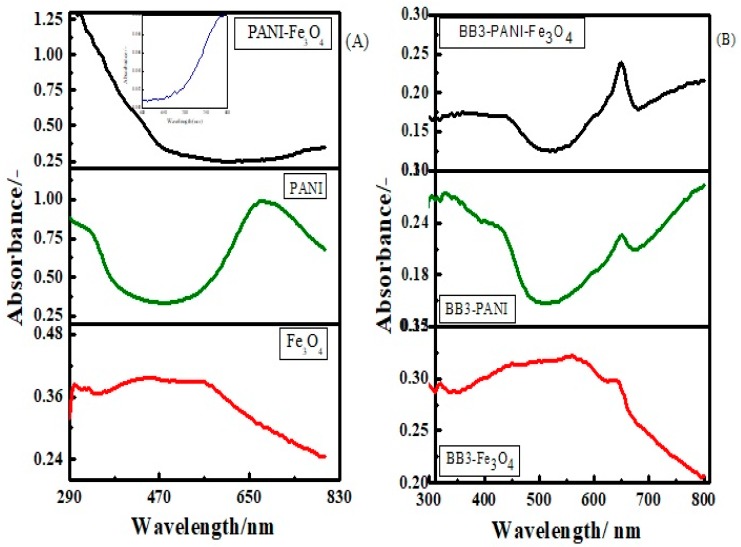
UV-Vis spectra of Fe_3_O_4_, PANI, and PANI/Fe_3_O_4_ composites (**A**) before and (**B**) after adsorption of BB3. The inset (A) shows the spectrum of PANI/Fe_3_O_4_ in the long wavelength region.

UV-Vis spectroscopy is widely used for studying optical properties of materials. UV-Visible spectra of Fe_3_O_4_, PANI, and PANI/Fe_3_O_4_ composites were recorded in ethanol and chloroform. Figure 2A shows the UV-Vis spectra of Fe_3_O_4_, PANI, and PANI/Fe_3_O_4_ composites before adsorption of BB3. In Fe_3_O_4_ spectrum, the band at 441.9 is due to the surface plasmon resonance effect (SPR). The surface plasmon resonance phenomenon occurs due to interactions between incident radiations and valence electrons of the metal atom in Fe_3_O_4_ and causes the valence electron of the metal to oscillate with the frequency of the electromagnetic source [57]. The other band at 570.7 nm arises due to the presence of DBSA moieties in the synthesized magnetic oxide particles, as reported earlier [58]. 

In the spectrum of PANI, the band at 325–338 nm is due to π-π* transitions of the benzenoid ring and the band at 660–680 nm is attributed to excitation of the quinoid ring [59]. The spectrum of PANI/Fe_3_O_4_ composites shows a small band at 441 nm due to doping of benzenoid amine with Fe_3_O_4_ particles, while the band at 770 nm is due to the change from polaron to bipolaron state, suggesting interactions between PANI and Fe_3_O_4_ materials, which is in close resemblance to the already reported results [60,61].

Figure 2B shows the UV-Vis spectra of Fe_3_O_4_, PANI, and PANI/Fe_3_O_4_ composites after adsorption of BB3, respectively. The appearance of absorption band at 647–651nm in all the spectra clearly indicates the adsorption of BB3 on Fe_3_O_4_, PANI, and PANI/Fe_3_O_4_ composites. As reported previously, BB3 gives a strong absorption band at 654 nm [62]. This absorption band is more intense in the spectrum of the composites as compared to the spectra of PANI and Fe_3_O_4_. The enhancement in the intensity of the absorption band of the composite around 650 nm shows strong interactions and adsorption capability of PANI/Fe_3_O_4_ composites towards BB3 as compared to pristine PANI and Fe_3_O_4_. 

#### 3.1.3. FTIR Spectroscopy

**Figure 3:**
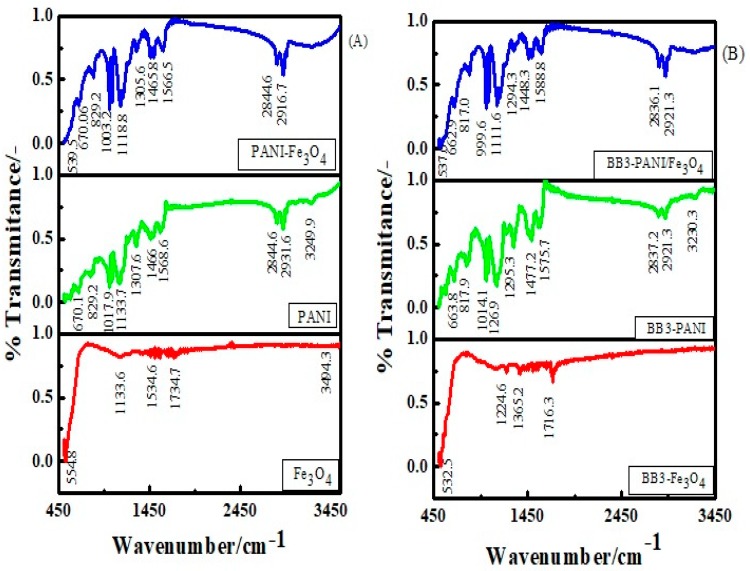
FTIR spectra of (**A**) Fe_3_O_4_, PANI, and PANI/Fe_3_O_4_ before and (**B**) after adsorption of BB3.

FTIR spectroscopy is used to study and identify organic, polymeric, and in some cases inorganic materials. Figure 3A shows FTIR spectra of Fe_3_O_4_, PANI, and PANI/Fe_3_O_4_ composites before adsorption of BB3. The details of FTIR signals associated with different types of vibrations are summarized in Table S2 of the Supplementary information.

A characteristic absorption band is observed at 554.8 cm^−1^ due to the stretching vibration of Fe–O bonds in the Fe_3_O_4_ spectrum. In an early study, stretching vibrations of Fe–O­­­­­­­­ bonds were reported at 560 cm^−1^ [63]. This shift in the Fe–O band towards lower frequency in the present study may be due to the presence of DBSA in the Fe_3_O_4_ particles. Peaks at 1133.6 and 1534.6 cm^−1^ correspond to CH_2_ bending modes of DBSA. Similarly, a weak peak at 3494.3 cm^−1^ is because of –OH stretching attached to the Fe_3_O_4_ surface and shows close resemblance to the already reported work [64]. Another weak band at 1734.7 cm^−1^ is assigned to residual NH_4_OH, as already reported elsewhere [65]. The peak at 554.8 cm^−1^ is due to stretching vibrations of Fe–O disappearing and a new peak at 539.5 cm^−1^ appearing, showing BB3 dye adsorbtion onto Fe_3_O_4_, as shown in Figure 3B. This is because of the interaction of oxygen present in the dye structure with Fe of Fe_3_O_4_. The appearance of more intense peaks at 1224.6 and 1365.7 in Figure 3B is also attributed to the adsorption of BB3 [66].

FTIR spectrum of PANI is also shown in Figure 3A. Peaks at 1568 cm^−1^ and 1466 cm^−1^ are due to C=C and C=N stretching vibrations of benzoinoid and quinoid rings, respectively. Phang and Kuramoto have reported the C=C and C=N stretching vibrations of PANI at 1572 and 1497 cm^−1^, respectively [54]. The bands at 1307.6 cm^−1^ are due to C–N•+ stretching of secondary aromatic amine of PANI doped with protic acid. The peak at 670.1 cm^−1^ shows out-of-plane bending vibrations of the C–H bond. The peak at 1017.9 cm^−1^ is assigned to –SO_3_H group of DBSA bonded to nitrogen of PANI. The bands at 1133.7 and 829.2 cm^−1^ are assigned to the aromatic C–H bending in-plane and out-of-plane deformation of C–H. The peaks at 2844.6, 2931.6, and 3249.9 cm^−1^ are assigned to N–H stretching vibrations of secondary amines. In the early research, such peaks appeared in the range of 3000–3500 cm^−1^ [67]. The shifting towards the low frequency range in the present work may be due to the presence of DBSA. After adsorption of BB3 dye, all these peaks shift towards high frequency, with a decrease in the intensity of peaks at 2844.6 and 2931.6 cm^−1^, as shown in the Figure 3B [50]. 

All these peaks appeared in the FTIR spectra of PANI/Fe_3_O_4_ composites, with a slight shift towards low frequency, as shown in Figure 3A. The shifting of absorption bands towards low frequency shows the existence of physical forces between PANI and Fe_3_O_4_. The band at 3249.9 cm^−1^ in the FTIR spectrum of PANI is replaced by a broad absorption plateau in the FTIR spectrum of PANI/Fe_3_O_4_ composites. The appearance of a very small peak at 539.5 cm^−1^, due to Fe–O bond stretching, shows the presence of Fe_3_O_4_ in the composite [68]. The absorption bands in the FTIR spectrum of PANI/Fe_3_O_4_ shift towards low frequency after adsorption of BB3, as was also observed in the spectra of PANI and Fe_3_O_4_, but the peaks are more intense in the former case, as shown in Figure 3B.

#### 3.1.4. EDX Spectroscopy

**Figure 4:**
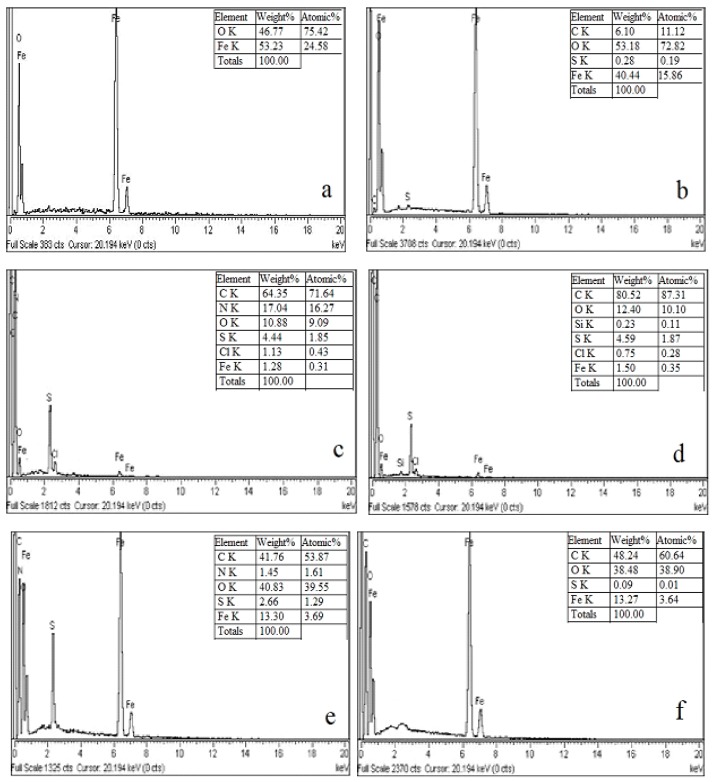
EDX of Fe_3_O_4_, PANI, and PANI/ Fe_3_O_4_ before (**a**,**c**,**e**) and after (**b**,**d**,**f**) adsorption of BB3.

EDX study is very important to analyze elemental composition of materials. Figure 4 shows the EDX spectra of Fe_3_O_4_, PANI, and PANI/Fe_3_O_4_ composites before and after adsorption of BB3 dye, respectively. The highest percentages of Fe and O is present in Fe_3_O_4_, which are 53.23 and 46.77 by weight, respectively (Figure 4a). Elsewhere, Fe and O contents were reported to be 41.6 and 41.56% [69]. After BB3 adsorption, Fe content was decreased to 40.44%, while O was increased to 53.18%. The increase in oxygen and appearance of carbon in the EDX spectrum (Figure 4b) are evidence of the adsorption of dye onto Fe_3_O_4_. 

The presence of C and N in the EDX spectra of PANI and PANI/ Fe_3_O_4_ composites indicates their formation. The weight percentages of C and N are 64.35 and 17.04 in PANI, respectively. Besides C and N, some other elements, such as Fe, O, S, and Cl, are also present in PANI texture. Their presence is attributed to the contribution from FeCl_3_ and DBSA, which were used as oxidant and emulsifying agents, respectively. The increase in the percentage weights of C and O indicates adsorption of BB3 on PANI, shown in Figure 4d. Figure 4e shows the EDX spectrum of the PANI/Fe_3_O_4_ composite. It contains 41.76 and 1.45% C and N, respectively, in addition to 13.27% Fe, indicating formation of the PANI/Fe_3_O_4_ composite. Like PANI, PANI/Fe_3_O_4_ composites also contain 2.66% S due to DBSA. In the EDX spectrum of the composite, the contents of both C and O increase after interaction with BB3 (Figure 4f), which suggests the adsorption of BB3 on the composite [70]. These observations support the results obtained through UV-Vis and FTIR spectroscopies. 

#### 3.1.5. XRD Study 

**Figure 5:**
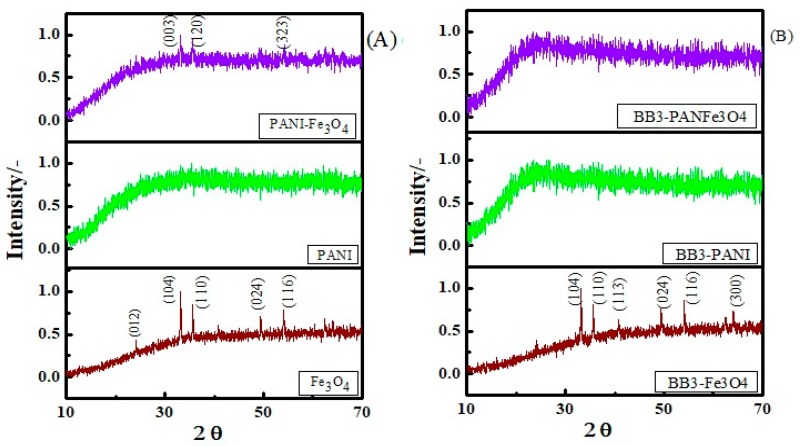
XRD of Fe_3_O_4_, PANI, and PANI/ Fe_3_O_4_ (**A**) before and (**B**) after adsorption of BB3.

X-ray diffraction is an important technique used to determine the structure and composition of synthesized materials. Figure 5A shows XRD patterns of Fe_3_O_4_, PANI, and PANI/Fe_3_O_4_ composites before adsorption of BB3. The characteristic diffraction peaks appeared at 2*θ* = 24.04°, 33.06°, 35.6°, 49.3°, 53.9°, and 62.7° in the XRD spectrum of Fe_3_O_4_, which indicates spinel cubic crystals of Fe_3_O_4_. The formation of a strong peak at 33.06° indicates the formation of Fe_3_O_4_. These peaks were matched with the standard cards on powder diffraction files-2 (PDF 89-598) and have close agreement [71]. After adsorption of BB3, the intensities of diffraction peaks decrease due to interactions between dye and Fe_3_O_4_ (Figure 5B) [72]. 

XRD spectrum (Figure 5A) of PANI shows its amorphous nature. No apparent change is observed in the spectrum of PANI after adsorption of BB3 (Figure 5B). Deshpande et al. [73] have reported a PANI film with amorphous shape. One can observe the presence of Fe_3_O_4_ in the PANI matrix due to diffraction peaks in the XRD spectrum of PANI/Fe_3_O_4_, but the intensities of these peaks are smaller than those in the spectrum of pure Fe_3_O_4_ particles, showing interaction between Fe_3_O_4_ and PANI. Obviously, the crystanality in the composites arises due to the presence of Fe_3_O_4_ particles. After adsorption of BB3 the peaks in the XRD spectrum of the composites simply disappeared. These observations indicate the strong overlaying layer of the dye on the surface of composites, thereby blunting the XRD peaks that were observed before adsorption of the dye [74].

#### 3.1.6. Surface Area Analysis

**Figure 6:**
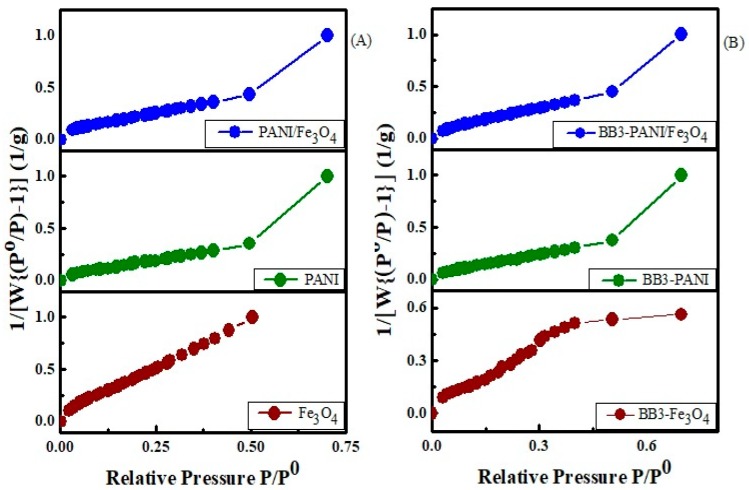
Surface area analysis of (**A**) Fe_3_O_4_, PANI, and PANI/ Fe_3_O_4_ before and (**B**) after adsorption of BB3.

Surface area analysis has a major role in the adsorption phenomenon. The surface areas of Fe_3_O_4_, PANI, and PANI/Fe_3_O_4_ composites before and after adsorption of BB3 were determined by adsorption–desorption of nitrogen gas through Brunauer–Emmett–Teller (BET) method (Figure 6) [75]. The obtained results are summarized in Table 1, which show that the surface areas of Fe_3_O_4_, PANI, and PANI/Fe_3_O_4_ composites before adsorption of BB3 are 65.818, 70.263, and 99.759 m^2^/g, respectively (Figure 6A). After adsorption of BB3, the surface areas of Fe_3_O_4_, PANI, and PANI/Fe_3_O_4_ composites decreased to 46.608, 46.698, and 53.196 m^2^/g, respectively (Figure 6B). The decrease in surface areas of Fe_3_O_4_, PANI, and PANI/Fe_3_O_4_ composites after adsorption of dye confirms that PANI/Fe_3_O_4_ composites can adsorb comparatively more dye than Fe_3_O_4_ and PANI. These results correlate to those obtained through SEM, XRD, EDX, and FTIR. 

Beside surface area, BET calculation can also be applied to determine the pore volume and average pore diameter, as shown in Table 1.

### 3.2. Equilibrium Study 

An equilibrium study is very valuable for understanding the interaction of BB3 with Fe_3_O_4_, PANI, and PANI/Fe_3_O_4_ composites. The adsorption data are shown in Table 2, which shows that the adsorption capacity of the dye on these materials increases as the concentration of dye increases. BB3 is a cationic dye and gets adsorbed on Fe_3_O_4_, PANI, and PANI/Fe_3_O_4_ composites from aqueous solution due to interactions with negative sites on the surface of the adsorbent. In the literature it has been explained that these binding sites are present (electron pair) on oxygen of Fe_3_O_4_ and nitrogen of amine and imine PANI and PANI/Fe_3_O_4_, which are capable of interacting with oppositely charged ions present in the dye [76]. The data obtained from the equilibrium study were fitted into Freundlich, Langmuir, Tempkin, and D-R adsorption isotherms for estimation of various adsorption parameters. 

**Figure 7:**
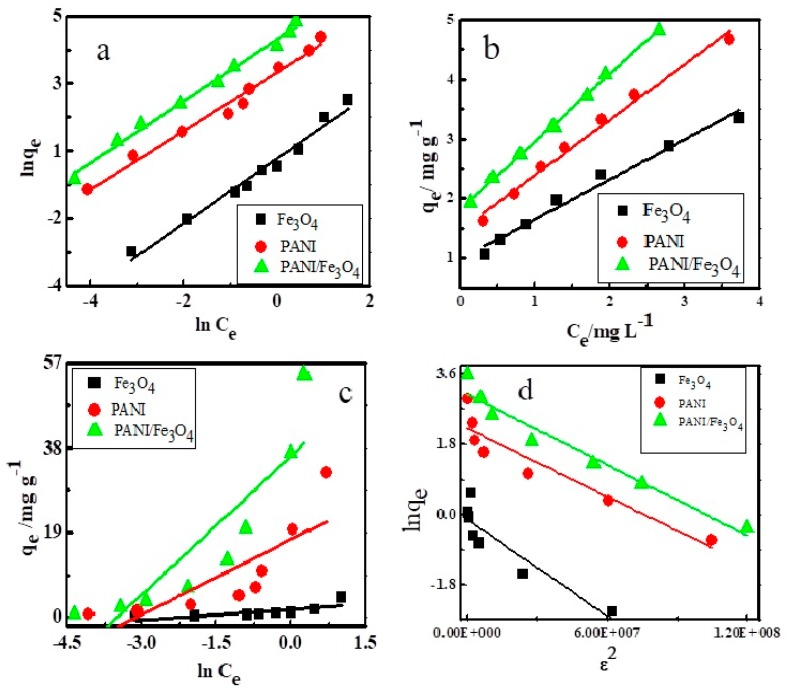
Adsorption of BB3 on Fe_3_O_4_, PANI, and PANI/Fe_3_O_4_. (**a**) Freundlich, (**b**) Langmuir, (**c**) Tempkin, and (**d**) D-R adsorption isotherms.

Freundlich adsorption equation is expressed by the following equation.

(2) $${\ln\;q}_{e}{=\mathrm{lnK}}_{f}+\frac{1}{n}{\ln\;C}_{e}$$

where q_e_ (mg/g) is the amount of dye adsorbed per gram of adsorbent, C_e_ (mg/L) is the concentration of dye at equilibrium, K_f_ is Freundlich isotherm constant, and n is the intensity of adsorbent. A plot of lnq_e_ vs. lnC_e_ is shown in Figure 7a.

From the value of the slope obtained from the Freundlich adsorption isotherm, it can be demonstrated whether adsorption is favorable or unfavorable, reversible or irreversible. It also explains whether the system is heterogeneous or not [77]. If 1/n > 1, adsorption is unfavorable at low concentration but favorable at high concentration; if 1/n < 1, adsorption is favorable over the entire range of concentrations and the system is heterogeneous. However, if 1/n = 1, then the system is homogenous [78]. The values of 1/n obtained from the Freundlich adsorption isotherm in the present study are 0.9593, 0.8673, and 0.9112 for adsorption of BB3 on Fe_3_O_4_, PANI, and PANI/Fe_3_O_4_ composites, respectively, as shown in Table 2. These values are in close resemblance with the literature showing that adsorption is favorable and heterogeneous. R^2^ values show that the Freundlich adsorption isotherm fits to the adsorption data for Fe_3_O_4_, PANI, and PANI/Fe_3_O_4_ composites.

The adsorption data were also analyzed through the Langmuir adsorption isotherm, which is expressed in the following equation.

(3) $$\frac{C_{e}}{q_{e}}=\frac{1}{q_{\max}K_{L}}+\frac{1}{q_{\max}C_{e}}$$

where q_max_ is the max adsorption capacity (mg/g), q_e_ is the amount of dye adsorbed at equilibrium (mg/g), Ce is the equilibrium adsorption concentration (mg/L), and K_L_ is the constant related to energy (Langmuir constant). From the Langmuir isotherm, R_L_ (dimensionless separating factor) is calculated by the following equation.

(4) $$R_{L}=\frac{1}{{(1+K}_{L}C_{i})}$$

From R_L_ value it can be demonstrated whether adsorption is favorable, unfavorable, reversible, or irreversible. If R_L_ value is less than one but more than zero (0 < R_L_ < 1) adsorption is favorable, but if 1 < R_L_ adsorption is unfavorable. If R_L_ = 0 adsorption is irreversible and R_L_ = 1 indicates that adsorption is reversible [79]. The adsorption data obtained through the Langmuir isotherm are given in Table 2, which show that the maximum adsorption capacities (q_max_) are 7.474, 47.977, and 78.13 mg/g for Fe_3_O_4_, PANI and PANI/Fe_3_O_4_ composites, respectively. The values of Langmuir constant (K_L_) and dimensionless separating constant (R_L_) for all the three types of adsorbents shows that adsorption of BB3 on Fe_3_O_4_, PANI, and PANI/Fe_3_O_4_ composites is monolayer and favorable. R^2^ values show that the Langmuir adsorption isotherm fits more closely to the adsorption data than the other isotherms. 

Tempkin adsorption isotherm, shown in the Equation (5), was also applied to explain the adsorption data.

(5) $$q_{e}{=\beta \mathrm{lnK}}_{T}{+\beta \mathrm{lnC}}_{e}$$

R^2^ values show that Tempkin isotherm does not fit very well to adsorption data as compared to Freundlich and Langmuir isotherms, but is still helpful in explaining the binding forces between adsorbents and adsorbate. K_T_ is the binding constant at equilibrium and corresponds to maximum binding energy [80]. Its values calculated from the intercept of plot q_e_ vs. lnC_e_ (Figure 7c) are 8.565, 22.26, and 33.04 L/g for Fe_3_O_4_, PANI, and PANI/Fe_3_O_4_ composites, respectively. These results show that there are strong binding forces between BB3 and PANI/Fe_3_O_4_ as compared to binding forces of dye with Fe_3_O_4_ and PANI, respectively. The value of constant β is related to the heat of adsorption in Equation (6)

(6) $$\beta \;=\frac{\mathrm{RT}}{b}$$

where b is Tempkin isotherm constant of binding energy (J/mol K). The negative sign of β values for all the three adsorbents shows that adsorption of BB3 on Fe_3_O_4_, PANI, and PANI/Fe_3_O_4_ composites is exothermic. 

The Dubinin-Radushkevitch (D-R) adsorption equation has also been successfully applied to the data obtained by plotting lnq_e_ vs. ε^2^, and is shown in Figure 7d. A linearized form of D.R adsorption equation is given below

(7) $${\mathrm{lnq}}_{e}{=\mathrm{lnq}}_{s}{-B\varepsilon }^{2}$$

where q_s_ is the theoretical monolayer saturation capacity (mg/g), B is the constant, called D-R model constant, and ε^2^ is the Polanyi potential, which is calculated by the Equation (8)

(8) $$\varepsilon =\mathrm{RTlog}(1+\frac{1}{C_{e}})$$

where R is the general gas constant and T is the absolute temperature. From the D-R model, energy of adsorption was calculated by Equation (9)

(9) $$E_{\mathrm{ad}}{}_{s}=\frac{1}{\sqrt{\left(1-2B\right)}}$$

In the literature it has been explained that for physical adsorption, the value of adsorption energy should be less than 40 kJ/mol [81]. Its value also tells about the route of adsorption through ion exchange process. In the early literature it has been explained that for ion exchange process the value of adsorption energy should be in the range of 8–16 kJ/mol. The values of q_s_ calculated from the linear plot of D-R isotherm are 0.888, 9.183, and 20.54 mg/g for Fe_3_O_4_, PANI, and PANI/Fe_3_O_4_ composites, respectively, showing that adsorption is physical. Similarly, values of (E_ads_), shown in Table 2, demonstrate that adsorption does not follow ion exchange process [82]. A comparison of the adsorption efficiency of the synthesized materials with those reported earlier is also provided in Table 3. 

### 3.3. Effect of Ionic Strength 

**Figure 8:**
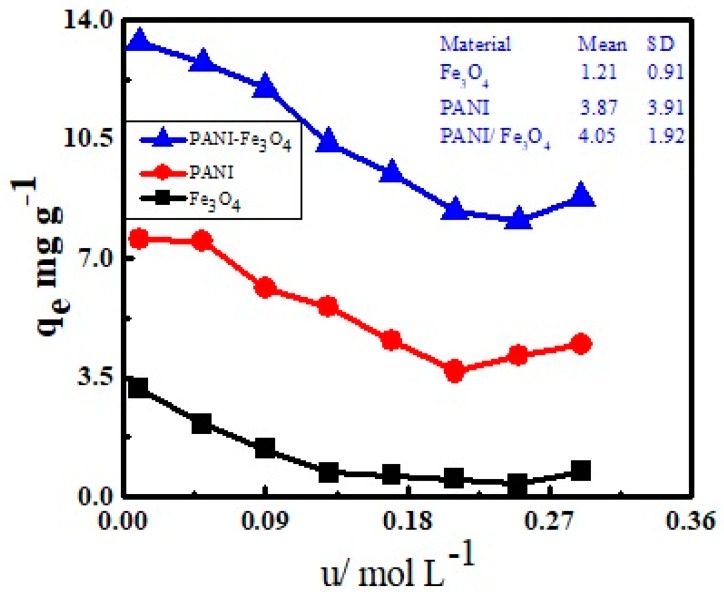
Effect of ionic strength on adsorption of BB3 on Fe_3_O_4_, PANI, and PANI/Fe_3_O_4_ composites.

Electrostatic interactions, such as ionic strength, greatly affect the surface properties of the adsorbent [91]. The effect of ionic strength on adsorption of BB3 (dye concentration 80 mg/L in 20 mL volume) on Fe_3_O_4_, PANI, and PANI/Fe_3_O_4_ was observed by adding sodium sulphate solution in the range of 0.01–0.3 mol dm^−3^. The obtained results (Figure 8) show that the adsorption capacities of Fe_3_O_4_, PANI, and PANI/Fe_3_O_4_ composites decrease as the concentration of salt (ionic strength) increases. The minimum dye adsorption on Fe_3_O_4_, PANI, and PANI/Fe_3_O_4_ was observed at 0.25, 0.21, and 0.25 ionic strengths, respectively. The competition of Na^+^ or SO_4_^2−^ ions with BB3 dye for active sites present on the surface of Fe_3_O_4_, PANI, and PANI/Fe_3_O_4_ might be a reason for the decrease in adsorption capability [71,92]. This competition is related to the interactions between hydrated ions and active sites of the adsorbent. Cations with a smaller hydrated radius occupy more active sites on the adsorbent, leading to stronger interaction with the adsorbent [93]. 

### 3.4. Effect of pH 

**Figure 9:**
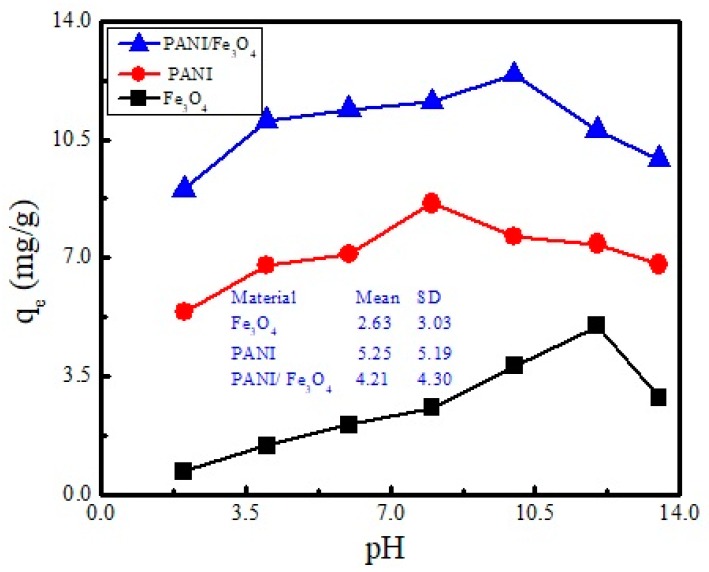
Effect of pH on adsorption of BB3 on Fe_3_O_4_, PANI, and PANI/Fe_3_O_4_ composite.

The pH of the solution plays a major role in the removal of adsorbates from aqueous solutions. Figure 9 shows the effect of pH on the adsorption of BB3 on Fe_3_O_4_, PANI, and PANI/Fe_3_O_4_. As BB3 is a cationic dye, at low pH the H^+^ ions compete with dye for active sites present on the surface of the adsorbent and protonate them. These active sites are Fe–O and –C–N groups. Similarly, the nitrogen and oxygen in the dye are also protonated. This causes electrostatic repulsion between dye and adsorbent, hence reducing adsorption [94]. As the pH of dye solutions increases, the adsorption increases and reaches a maximum for Fe_3_O_4_, PANI, and PANI/Fe_3_O_4_ composites when the pH of the dye solution is 12, 8, and 10, respectively. At high pH de-protonation of Fe–OH and –C–N–H groups occurs, resulting in negatively charged sites, such as Fe–O^−^ and –C–N^−^, which have stronger interactions with dye. Figure 9 also indicates that after optimum pH, adsorption once again decreases. This may be due to hydroxylation of active sites of adsorbents [95]. 

### 3.5. Effect of Contact Time and Temperature

**Figure 10:**
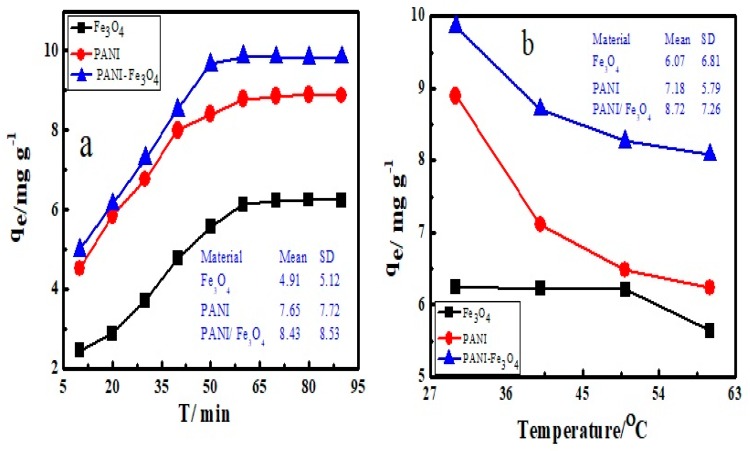
Adsorption of BB3 at (**a**) different time intervals and (**b**) temperature on Fe_3_O_4_, PANI, and PANI/Fe_3_O_4_ composites.

Contact time and temperature are also important parameters for explaining the adsorption phenomenon. The adsorption of BB3 on Fe_3_O_4_, PANI, and PANI/Fe_3_O_4_ composites as a function of time is shown in Figure 10a, which shows that the adsorption increases with the passage of time. This figure also shows that initially adsorption is fast and contributes significantly to the equilibrium, but as the time passes, the adsorption slows down and its contribution to equilibrium decreases. This is due to filling of active sites on the surface of the adsorbent by the molecules of dye, and gradually adsorption becomes less effective. At this time, a dynamic equilibrium is established between the amount of dye adsorbed and desorbed from the adsorbent. This time is termed “equilibrium time” and the dye adsorbed at the equilibrium time is referred to as the maximum adsorption capacity of the adsorbent. It is evident from Figure 10a that the equilibrium time of adsorption is reached for Fe_3_O_4_, PANI, and PANI/Fe_3_O_4_ composites within 50 to 60 min [96]. Figure 10b shows that adsorption of BB3 on PANI and PANI/Fe_3_O_4_ composites is maximal at 30 °C and decreases beyond this temperature, indicating exothermic behavior. 

### 3.6. Effect of Adsorbent Dose

**Figure 11:**
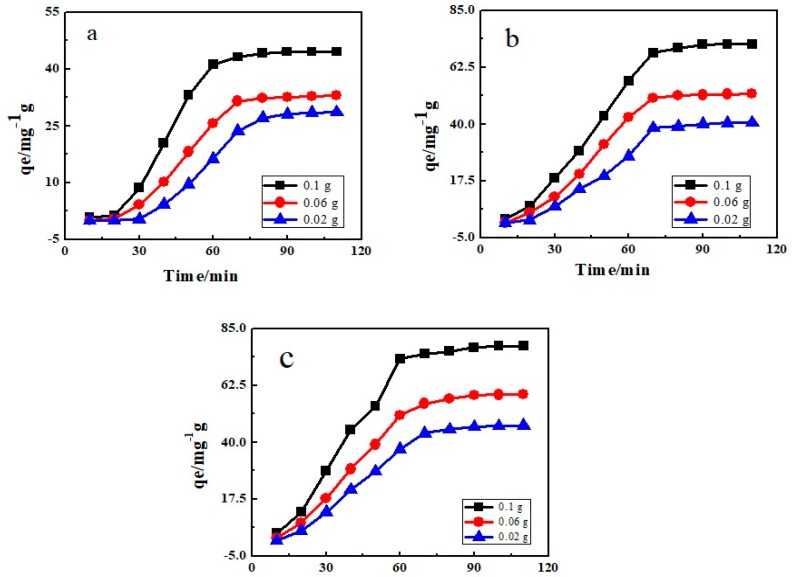
Effect of adsorbent dose for (**a**) Fe_3_O_4_, (**b**) PANI, and (**c**) PANI/ Fe_3_O_4_ composite on adsorption of BB3.

The effect of adsorbent dose on adsorption of BB3 (50 mg/L) is studied with different amounts (0.02 g, 0.06 g, and 0.1 g) of Fe_3_O_4_, PANI, and PANI/Fe_3_O_4_ composites, respectively. The results are shown in the Figure 11, which shows that amount of adsorption of BB3 increases as the amount of adsorbent increases. This shows that as the amount of adsorbent increases, more active sites are available for the adsorption of dye, which results in more interactions between dye and adsorbent. The figure shows that the adsorption capacity of PANI/Fe_3_O_4_ composites is more than Fe_3_O_4_ and PANI. 

### 3.7. Kinetic Study 

**Figure 12:**
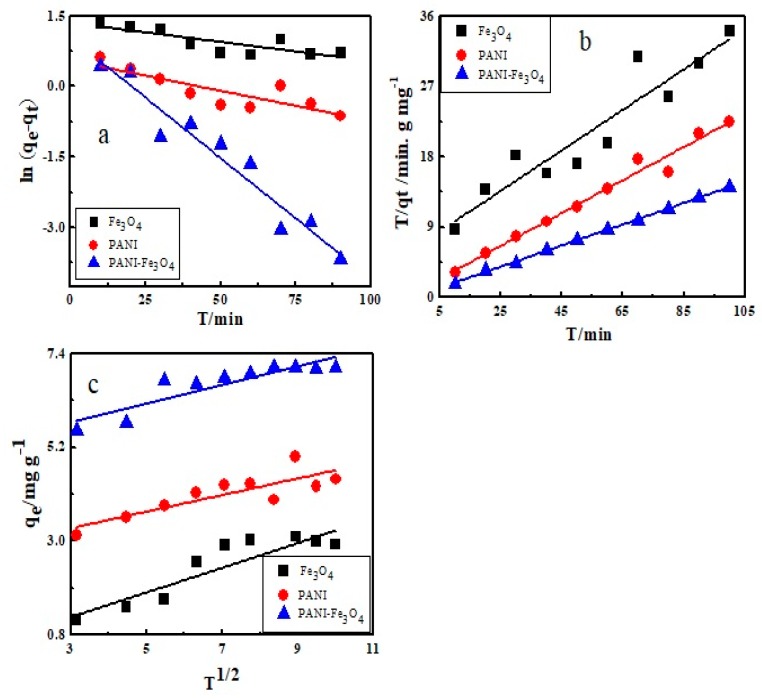
Kinetics models: (**a**) First order and (**b**) second order kinetics, (**c**) intra-particle diffusion model of adsorption of BB3 dye on Fe_3_O_4_, PANI, and PANI/Fe_3_O_4_ composite.

Kinetic study is very important to explain the adsorption phenomenon. The data obtained from adsorption of BB3 dye were analyzed through Lagergren’s pseudo first order, Ho and McKay’s pseudo second order, and Weber and Morris’s intra particle diffusion models by using Equations (10)–(12).

(10) $${\log(q}_{e}{-q}_{t}{)=\mathrm{logq}}_{e}-\frac{K_{1}t}{2.303}$$

(11) $$\frac{t}{q_{t}}=\frac{1}{K_{2}q_{e}{}^{2}}+\frac{t}{q_{e}}$$

q_t_ = k_d_.t^1/2^ + C (12)

where q_e_ and q_t_ are the amount of dye adsorbed (mg g^-1^) at equilibrium and at time t, K_1_, K_2_, and K_d_ are rate constant of pseudo first order (min^−1^), pseudo second order (g mg^−1^ min^−1^), and intra-particle diffusion models (g mg^−1^ min^−1/2^), respectively. C (mg g^-1^) is the constant and t is the time in minutes. Figure 12a–c shows the fitted curves of pseudo first order, pseudo second order, and intra-particle diffusion models for BB3 adsorbed on Fe_3_O_4_, PANI, and PANI/Fe_3_O_4_ composites, respectively. The kinetics data obtained are shown in Table 4. The correlation factor (R^2^) indicates that the pseudo second order kinetic model fits more closely to data as compared to the pseudo first order and intra-particle diffusion models. Values of rate constant indicate that as the temperature increases, rate of adsorption decreases [77,78].

### 3.8. Mechanism of Adsorption

**Scheme 3:**
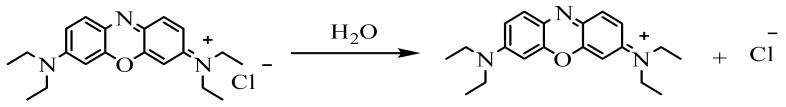
Dissociation of BB3 in a positively-charged complex cation and negatively charged chloride ion into.

Actually, many factors, such as structure and charge on dye, surface of adsorbent, hydrophilic, and hydrophobic properties, electrostatic interaction, and physical forces, such as hydrogen bonding and dipole-dipole interaction, affect the adsorption of BB3 on PANI/Fe_3_O_4_ composites. Therefore, different mechanisms can be proposed for the adsorption of BB3 on Fe_3_O_4_, PANI, and PANI/ Fe_3_O_4_ composites. When BB3 is added to water, it dissociates in a positively-charged complex cation and negatively charged chloride ion as shown below (Scheme 3).

There is a possibility of H-bonding between amine and imine groups of PANI/Fe_3_O_4_ with nitrogen and oxygen present in the BB3 structure. Similarly, the surface hydroxyl groups of Fe_3_O_4_ may also form H-bonds with dye molecules [97].

**Scheme 4:**
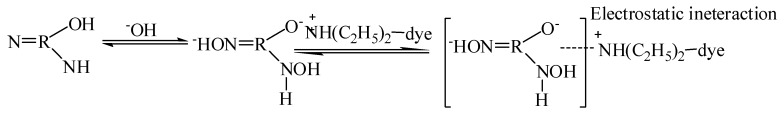
Adsorption behavior of BB3 on PANI/Fe_3_O_4_ in basic medium.

There may exist Vander Waal’s interaction between hydrophobic parts of the dye and hydrophobic parts of the PANI/Fe_3_O_4_ composite, because the nonpolar groups have a tendency to associate in aqueous solution. Another possibility is the existence of electrostatic interaction between positively-charged nitrogen present in the dye structure and a lone pair present on the nitrogen of amine and imine group of PANI and PANI/Fe_3_O_4_ [98]. The adsorption behavior of BB3 on PANI/Fe_3_O_4_ in basic medium is shown as the following (Scheme 4).

Where R represents the non-polar part of the PANI/Fe_3_O_4_ with = NH, −NH_2_ of PANI, and −OH group of Fe_3_O_4_.

During the adsorption process the amount of energy released compensates for the entropy change of adsorbed molecules and depends upon the forces between adsorbent and adsorbate molecules; the stronger the force, the more energy will be released. The energy released during the adsorption process for H-bond is (2–40 kJ/mol), dipole-dipole interaction is (2–29 kJ/mol), Vander Waals forces is (4–10 kJ/mol), and is about 5 kJ/mol for hydrophobic forces, and more than 60 kJ/mol for electrostatic interaction [99]. In the present study the enthalpy change are −32.84, −62.93, and −74.26 kJ/mol when BB3 adsorbs on Fe_3_O_4,_ PANI, and PANI/Fe_3_O_4_, respectively.

### 3.9. Calculation of Thermodynamic Parameters 

Thermodynamic parameters, such as activation energy, Gibb’s free energy change, enthalpy change, and entropy change, are helpful to explain the nature of adsorption. Activation energy is calculated by Arrhenius equation, shown below.

k = Aexp^(−Ea/RT)^ (13)

where Ea is the activation energy, T is the absolute temperature, A is the Arrhenius constant, and k is the rate constant. Gibb’s Free energy change is calculated by the following equation.

(14) $$\Delta G=-\mathrm{RTln}\frac{q_{e}}{C_{e}}$$

**Figure 13:**
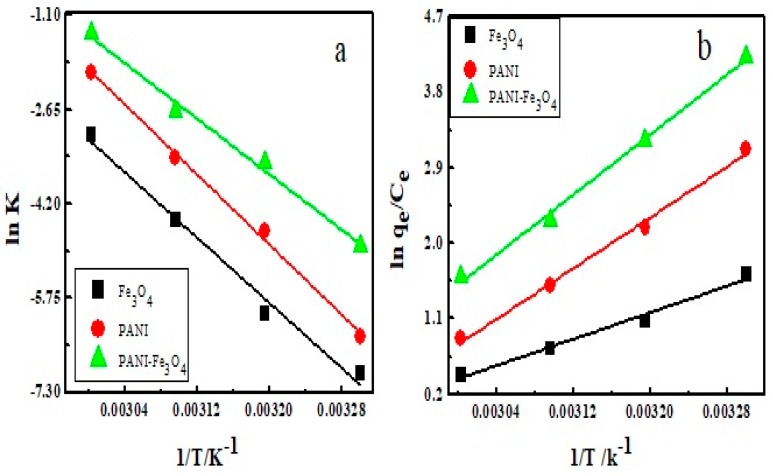
(**a**) Arrhenius plot for calculation of activation energy. (**b**) The van’t Hoff plot for calculation of enthalpy and entropy.

Enthalpy change and entropy change are calculated by Van’t Hoff equation by plotting the lnq_e_/C_e_ vs. 1/T, as given below.

(15) $$\ln\frac{q_{e}}{C_{e}}=\frac{\Delta S}{R}-\frac{\Delta H}{\mathrm{RT}}$$

where ΔH is the enthalpy change and ΔS is the change in entropy, and T is the absolute temperature. Figure 13a shows the Arrhenius plot, obtained by plotting lnK_2_ vs. 1/T after adsorption of BB3. From the slope the activation energy values of adsorption of BB3 were to found to be 11.14, 11.97, and 09.94 kJ/mol, respectively, which indicate that adsorption is physical and is a diffusion control process (Table 5) [100]. The value of enthalpy change is also helpful in explaining the adsorption phenomenon. It was reported that enthalpy change in the range of 84–420 kJ/mol suggests chemical interaction between dye and adsorbent (chemisorption), while its value below 84 kJ/mol indicates physical adsorption [95]. The values of enthalpy change in the present work, as shown in Table 5, are −32.84, −62.93, and −74.26 kJ/mol for the adsorption of BB3 on Fe_3_O_4,_ PANI, and PANI/Fe_3_O_4_ composite, respectively, thereby confirming the physical process. The negative sign of ΔH indicates that adsorption is exothermic. The ΔG value is also helpful in explaining the adsorption phenomenon, it explains the spontaneity and non-spontaneity of adsorption. The negative sign for ΔG shown in Table 5 indicates that adsorption is exothermic and spontaneous. The ΔG values in the range of −20 to 0 kJ/mol show physiosorption, and from −400 to −80 kJ/mol show chemisorption [101,102]. The ΔG values for Fe_3_O_4,_ PANI, and PANI/Fe_3_O_4_ composite used as adsorbents are −04.05, −07.78, and −10.63 kJ/mol, respectively, which suggest that adsorption of BB3 dye on all the three adsorbents is physical, exothermic, and spontaneous [103]. These observations strongly correlate with the data presented in Section 3.5 for temperature effect on the absorption phenomenon.

## 4. Conclusions

PANI/Fe_3_O_4_ composites, whose syntheses were confirmed through various spectroscopic techniques, such as SEM, FTIR, EDX, UV, and XRD, can effectively be utilized as adsorbents for removal of BB3 (cationic dye) from aqueous solution. It was envisaged that the synergy between PANI and magnetite would impart promising properties onto the composite material, as a high amount of dye (78.13 mg/g) was adsorbed on PANI/Fe_3_O_4_ composites in comparison to that adsorbed for Fe_3_O_4_ (7.474 mg/g) and PANI (47.977). The enhanced adsorption capability of the composites is attributed to the increase in surface area and pore volume of the hybrid materials. The adsorption followed pseudo second order kinetics, with R^2^ values of 0.873, 0.979, and 0.999 for Fe_3_O_4_, PANI, and PANI/Fe_3_O_4_ composites, respectively. The activation energy, enthalpy, Gibbs free energy changes, and entropy changes were found to be 11.14, −32.84, −04.05, and −0.095 kJ/mol for Fe_3_O_4_, 11.97, −62.93, −07.78, and −0.18 kJ/mol for PANI, and 09.94, −74.26, −10.63, and −0.210 kJ/mol for PANI/Fe_3_O_4_, respectively, indicating the spontaneous and exothermic nature of the adsorption process. The Langmuir adsorption isotherm model fitted more closely to the data. The adsorption was greater in basic medium than in acidic medium. The adsorption was well-described by the pseudo second order kinetic model. Thermodynamically, adsorption is proven to be exothermic and spontaneous. 

## Figures and Tables

**Table 1:** Surface area and Barrett, Joyner, and Halenda (BJH) para meters of Fe_3_O_4_, PANI, and PANI/Fe_3_O_4_ composites before and after adsorption of BB3 dye.

Observations	Sample	BJH Average Pore Radius (Å)	BJH Pore Volume (cc/g)	Surface Area (m^2^/g)
**Before adsorption**	Fe_3_O_4_	14.879	0.033	65.818
	PANI	15.500	0.021	70.263
	PANI/Fe_3_O_4_	14.951	0.062	99.759
**After adsorption**	Fe_3_O_4_	14.864	0.023	46.608
	PANI	14.822	0.020	46.698
	PANI/Fe_3_O_4_	14.944	0.046	53.196

**Table 2:** Parameters calculated from adsorption isotherm models applied for adsorption of BB3 on Fe_3_O_4_, PANI, and PANI/Fe_3_O_4_ composites.

Adsorbents	Freundlich		Langmuir		Tempkin		D-R	
**Fe_3_O_4_**	1/n	0.9593	q_max_	7.474	β	−0.8096	q_s_	0.888
	K_f_	1.312	K_L_	0.0911	K_T_	8.565	E_ads_	0.899
	R^2^	0.9755	R_L_	0.1210	R^2^	0.5048	R^2^	0.8036
	-	-	R^2^	0.9788	-	-	-	-
**PANI**	1/n	0.8673	q_max_	47.977	β	−5.626	q_s_	9.183
	K_f_	16.912	K_L_	0.0141	K_T_	22.26	E_ads_	0.999
	R^2^	0.9797	R_L_	0.4710	R^2^	0.6196	R^2^	0.8620
	-	-	R^2^	0.9849	-	-	-	-
**PANI/Fe_3_O_4_**	1/n	0.9112	q_max_	78.13	β	−10.372	q_s_	20.54
	K_f_	44.719	K_L_	0.0071	K_T_	33.04	E_ads_	0.897
	R^2^	0.9911	R_L_	0.6410	R^2^	0.7514	R^2^	0.9437
	-	-	R^2^	0.9985	-	-	-	-

**Table 3:** Comparative adsorption of BB3 on Fe_3_O_4_, PANI, and PANI/Fe_3_O_4_ with other adsorbents.

Adsorbents	pH	T (°C)	Maximum Adsorption (mg/g)	Refs.
Aleppo pine-tree sawdust	7	20	65.4	[83]
Ethylenediamine modified rice husk	4.7	25	3.29	[84]
Wood activated Charcoal	7	10–50	0.59–0.64	[85]
Quartinized sugarcane bagass	6–8	20	37.59	[86]
Activated sludge biomass	-	20	36.5	[87]
Palm fruit bunch particles	-	-	91.33	[88]
CM-60 weak acid acrylic resin	5.5	17–50	34.36–59.53	[89]
Activated Carbon	8	32	406	[90]
Fe_3_O_4_	12	30	7.474	Present study
PANI	8	30	47.97	Present study
PANI/Fe_3_O_4_	10	30	78.13	Present study

**Table 4:** Parameters of Kinetics models of BB3 adsorption onto Fe_3_O_4_, PANI, and PANI/Fe_3_O_4_ composite.

Pseudo 1st Order				Pseudo 2nd Order			Intra Particle Diffusion				
Adsorbents	K_1_ (min^−1^)	q_e_ (mg g^−1^)	R^2^	K_2_ (g mg^−1^ min^−1^)	q_e_ (mg g^−1^)	R^2^		K_d_ (g mg^−1^ min^−1/2^)		C (mg g^−1^)	R^2^
**Fe_3_O_4_**	−0.0081	3.859	0.644	0.259	7.145	0.873	0.2887		0.353		0.745
**PANI**	−0.0128	1.733	0.688	0.211	44.50	0.979	0.1946		2.712		0.679
**PANI/Fe_3_O_4_**	−0.0513	2.869	0.932	0.136	76.71	0.999	0.2192		5.122		0.777

**Table 5:** Thermodynamic parameters of adsorption of BB3 on Fe_3_O_4_, PANI, and PANI/Fe_3_O_4_ composite.

Adsorbents	(kJ/mol)	(kJ/mol)	(kJ/(mol·K))	Ea (kJ/mol)
**Fe_3_O_4_**	−04.05	−32.84	−0.095	11.14
**PANI**	−07.78	−62.93	−0.182	11.97
**PANI/Fe_3_O_4_**	−10.63	−74.26	−0.210	09.94

## Supplementary Materials

The following are available online at https://www.mdpi.com/1996-1944/12/11/1764/s1, Table S1: Comparison of different synthesis methods for PANI/iron oxide and their use as adsorbent for removal of various dyes, Table S2: Summery of FTIR absorption bands.
