# Supplementary material for: Basic Blue Dye Adsorption from Water Using Polyaniline/Magnetite (Fe3O4) Composites: Kinetic and Thermodynamic Aspects
Source: Materials (Basel). 2019 May 30;12(11):1764. doi: 10.3390/ma12111764 (PMC6600751; doi:10.3390/ma12111764)
Supplement: Supplementary file 1 [file materials-12-01764-s001.pdf]

materials

MDPI

Article

Basic Blue Dye Adsorption from Water using Polyaniline/Magnetite(Fe_3O_4) Composites: Kinetic and Thermodynamic Aspects

Amir Muhammad [1], Anwar-ul-Haq Ali Shah [1,*], Salma Bilal [2,3,*] and Gul Rahman [1]

1. Institute of Chemical Sciences, University of Peshawar, Peshawar 25120, Pakistan; amirics2015@gmail.com (A.M.); gul_rahman47@uop.edu.pk (G.R.)
2. National Centre of Excellence in Physical Chemistry, University of Peshawar, Peshawar 25120, Pakistan
3. TU Braunschweig Institute of Energy and Process Systems Engineering, Franz-Liszt-Straße 35, 38106 Braunschweig, Germany

* Correspondence: anwarulhaqalishah@uop.edu.pk (A.A.S.); s.bilal@tu-braunschweig.de or dresalmabilal@gmail.com (S.B.); Tel.: 0092-919216652 (A.A.S); 0049-531-39163651 or 0092-919216766 (S.B.)

Supplementary Information

Table S1. Comparison of different synthesis methods for PANI/iron oxide and their use as adsorbent for removal of various dyes.

Material	Raw Materials	Methodology	Dye removed from water	Adsorption isotherm used	Ref.
**Fe_3O_4**	$FeSO_4{\cdot}7H_2O$, NaOH	Co-precipiation	-	-	[1]
**PANI/Fe_3O_4**	Aniline as monomer, Fe_3O_4, HCl as dopend, dodecyl pyridinum bromide as surfactant and potassium persulfonate as oxidant	Chemical oxidation methods	amido black 10B dye which is anionic dye	Freudlich, Langmuir adsorption isotherm models	
**PANI/Fe_3O_4**	Aniline monome, Poly(sodium 4-styrenesulfonate) as surfactant, H_2O_2 as oxidant	Chemical oxidation method	No adsorption study	Nil	[2]
**Fe_3O_4**	$Fe(NO_3)$ $39H_2O$, $FeSO_4$ $6H_2O$ and NH_4OH	Co-precipitation method	No adsortion study	Nil	[3]
**PANI/Fe_3O_4**	Aniline monomer, Fe_3O_4 particles, ammonium persulfate as oxidant	Chemical oxidation method	Photocatalytic degradation of acid violet 19 dye	Nil	
**Fe_3O_4**	$FeCl_3$ $6H_2O$ $FeSO_4$ $7H_2O$ DBSA, NH_4OH	Co-precipitation method	Adsorbed Basic Blue 3 dye	Freundlich, Langmuir, Tempkin and D-R adsorption isother	Present Study
**PANI**	Aniline monomer, DBSA surfactant as well dopend, $FeCl_3.6H_2O$ As oxidant	Chemical oxidation method	Adsorbed Basic Blue 3 dye	Freundlich, Langmuir, Tempkin and D-R adsorption isother	
**PANI/Fe_3O_4**	Aniline monomer, DBSA surfactant as well dopend, $FeCl_3$ $6H_2O$ As oxidant, Fe_3O_4 particles	Chemical oxidation method	Adsorbed Basic Blue 3 dye	Freundlich, Langmuir, Tempkin and D-R adsorption isother	

Table S2. Summary of FTIR absorption bands.

Adsorbents	Absorption bands (cm^{-1})		Assignments	Ref.
	Before adsorption	After adsorption		
Fe_3O_4	554.8	539.5	Fe–O stretching vib	[4]
	1133.6	1224.6	}–CH_2– bending due to DBSA	[5]
	1534.6	1365.7		
	3494.3	-	–OH stretching	[5]
	1734.7	1763	Residual NH_4OH	[6]
PANI	1568	1575.7	C=C stretching vib of benzenoid	[7]
	1466	1477.2	C=N stretching vib of quinoid rings	[7]
	1307.6	1285.3	C–N stretching vib of sec. aromatic amine	[8]
	670.1	663.8	C–H out plane bending vib.	[8]
	1017.9	1014.1	–SO_3H group of DBSA	[8]
	1133.7	1269	C–H bending in plane	[8]
	829.2	817.9	C–H out plane deformation	[8]
	3249.9	3249.9	}N–H sec. amine	[9]
	2931.6	2921.3		
	2844.6	2837.2		
PANI/ Fe_3O_4	539.5	537.6	Fe–O stretching vib	[10]
	670.06	662.9	C–H out plane bending vib	[10]
	829.2	811.0	C–H out plane deformation	[8]
	1003.2	999.6	–SO3H group of DBSA	[9]
	1118.8	1111.6	C-H bending inplane	[9]
	1305.6	1294.3	C–N stretching vib. Of sec. aromatic amine	[9]
	1465.8	1448.3	C=N stretching vib. Of quinoid ring	[9]
	1566.5	1588.8	C=C stretching vib of benzoinoid	[9]
	2916.7	2921.3	}N–H sec amine	[9]
	2844.6	2836.1		

References

1. Ahmad, R.; Kumar, R. Conducting Polyaniline/Iron Oxide Composite: A Novel Adsorbent for the Removal of Amido Black 10B. *J. Chem. Eng. Data* **2010**, *55*, 3489–3493. doi:10.1021/je1001686.
2. Yang, C.; Du, J.; Peng, Q.; Qiao, R.; Chen, W.; Xu, C.; Shuai, Z.; Gao, M. Polyaniline/Fe_3O_4 Nanoparticle Composite: Synthesis and Reaction Mechanism. *J. Phys. Chem. B* **2009**, *113*, 5052–5058. doi:10.1021/jp811125k.
3. Patil, M.R.; Khairnar, S.D.; Shrivastava, V.S. Synthesis, characterisation of polyaniline–Fe_3O_4 magnetic nanocomposite and its application for removal of an acid violet 19 dye. *Appl. Nanosci.* **2016**, *6*, 495–502. doi:10.1007/s13204-015-0465-z.
4. Roychowdhury, A.; Pati, S.P.; Mishra, A.K.; Kumar, S.; Das, D. Magnetically addressable fluorescent Fe_3O_4/ZnO nanocomposites: Structural, optical and magnetization studies. *J. Phys. Chem. Solids* **2013**, *74*, 811–818. doi:10.1016/j.jpcs.2013.01.012.
5. Gholivand, M.B.; Yamini, Y.; Dayeni, M.; Seidi, S. Removal of Methylene Blue and Neutral Red from Aqueous Solutions by Surfactant-Modified Magnetic Nanoparticles as Highly Efficient Adsorbent. *Environ. Prog. Sustain. Energy* **2015**, *34*, 1683–1693. doi:10.1002/ep.12174.
6. Khoshnevisan, K.; Barkhi, M.; Zare, D.; Davoodi, D.; Tabatabaei, M. Preparation and Characterization of CTAB-Coated Fe_3O_4 Nanoparticles. *Nano-Metal Chem.* **2012**, *42*, 644–648. doi:10.1080/15533174.2011.614997.
7. Cao, C.; Xiao, L.; Chen, C.; Shi, X.; Cao, Q.; Gao, L. In situ preparation of magnetic Fe_3O_4/chitosan nanoparticles via a novel reduction–precipitation method and their application in adsorption of reactive azo dye. *Powder Technol.* **2014**, *260*, 90–97. doi:10.1016/j.powtec.2014.03.025.
8. Wai, P.B.S.; Kuramoto, N. Development and Investigation of Polyaniline Micro/nanocomposites that Possess Moderate Conductivity, Dielectric and Magnetic Properties. *Polymer* **2008**, *40*, 25–32. doi:10.1295/polymj.PJ2007049.
9. Ayad, M.; Hefnawy, G.E.; Zaghlol, S. Facile synthesis of polyaniline nanoparticles; its adsorption behavior. *J. Chem. Eng.* **2013**, *217*, 460–465. doi:10.1016/j.cej.2012.11.099.

10. Shena, J.; Shahida, S.; Amuraa, I.; Sarihana, A.; Tiana, M.; Emanuelsson, E.A. Enhanced adsorption of cationic and anionic dyes from aqueous solutions by polyacid doped polyaniline. *Synth. Met.* **2018**, *245*, 151–159. doi:10.1016/j.synthmet.2018.08.015.

© 2019 by the authors. Licensee MDPI, Basel, Switzerland. This article is an open access article distributed under the terms and conditions of the Creative Commons Attribution (CC BY) license (http://creativecommons.org/licenses/by/4.0/).
